# COVID-19 Preventive Behaviors and Health Literacy, Information Evaluation, and Decision-making Skills in Japanese Adults: Cross-sectional Survey Study

**DOI:** 10.2196/34966

**Published:** 2022-01-24

**Authors:** Kazuhiro Nakayama, Yuki Yonekura, Hitomi Danya, Kanako Hagiwara

**Affiliations:** 1 Department of Nursing Informatics Graduate School of Nursing Science St. Luke’s International University Tokyo Japan

**Keywords:** coronavirus, COVID-19, health literacy, health information, decision-making, health promotion, prevention, behavior, survey, evaluation

## Abstract

**Background:**

Health literacy is important for the prevention of COVID-19 transmission. Research in Japan shows that health literacy is related to skills in evaluating information and decision-making (skills that are not necessarily limited to information about health). Such basic skills are important, particularly when individuals encounter new health issues for which there is insufficient evidence.

**Objective:**

We aimed to determine the extent to which COVID-19 preventive behaviors were associated with health literacy and skills in evaluating information and making decisions.

**Methods:**

A web-based questionnaire survey was conducted using a Japanese internet research company. The measures comprised 8 items on COVID-19 preventive behaviors, health literacy items (European Health Literacy Survey Questionnaire), 5 items on information evaluation, and 4 items on decision-making process. Pearson correlations between these variables were calculated. Multivariable analyses were also conducted using the COVID-19 preventive behavior score as a dependent variable.

**Results:**

A total of 3914 valid responses were received.COVID-19 preventive behaviors were significantly correlated with health literacy (r=0.23), information evaluation (r=0.24), and decision-making process (r=0.30). Standardized regression coefficients (health literacy: β=.11; information evaluation: β=.13; decision-making: β=.18) showed that decision-making process contributed the most.

**Conclusions:**

Although comprehensive health literacy is necessary for COVID-19 preventive behaviors, the skills to evaluate a wide range of information and to make appropriate decisions are no less important. Opportunities for people to acquire these skills should be available at all times.

## Introduction

Health literacy is as important for the prevention of COVID-19 virus transmission as it is for the prevention of noncommunicable diseases [1]. Messages and materials about COVID-19 incorporate unusual vocabulary and phrases, and the number of COVID-19–related cases and deaths may be interpreted differently by people within a particular region or country [2]. Without adequate health literacy, people are unable to distinguish fact from fiction, and their behavior may be affected by unreliable information [3]. Low health literacy and information gaps may contribute substantially to the rapid spread of fear and anxiety [4]. Conversely, high health literacy can “flatten the curve” (ie, slow infection rates) of COVID-19 infections [5].

Research shows that health literacy, which has been measured in a variety of ways for a diverse range of topics, is associated with knowledge and behavior related to COVID-19: participants with less comprehensive health literacy expressed more confusion about COVID-19–related information [6], and a study [7] on digital health literacy related to COVID-19 found that college students who were better at evaluating reliability and determining the relevance of the information that they received used more reliable public websites rather than search engines or social media.

Similarly, adolescents with higher comprehensive health literacy who were asked to respond to the pandemic situation were more likely to be knowledgeable, more likely to wash their hands, less likely to socialize with friends, and more likely to report higher health-related quality of life [8]. Higher levels of infectious disease–specific health literacy or COVID-19–related health literacy are associated with greater implementation of COVID-19 preventive behaviors [9,10].

Several studies [11-13] have investigated the relationship between health literacy not associated with COVID-19 and COVID-19–related behaviors, mental health, and quality of life. In a study [11] of the Australian general public using the Single Item Literacy Screener that reported differences in knowledge, attitudes, and behaviors, participants with lower health literacy had more difficulty understanding COVID-19 symptoms, infection-prevention behaviors, and government information. In a study [13] using the comprehensive health literacy scale developed by the European Health Literacy Project [12], chronically ill patients with greater health literacy engaged more in COVID-19 preventive behaviors [13]. Similarly, in a study [14] that assessed comprehensive health literacy in an outpatient setting, greater health literacy was associated with lower depression and greater health-related quality of life, even when COVID-19 symptoms were suspected. In addition, a study [15] of medical students found that higher comprehensive health literacy was associated with lower fear of COVID-19 [15]. Among health care workers, higher comprehensive health literacy is associated with better infection prevention and control procedures, healthier lifestyles [16], and better mental health and quality of life [17], even during the COVID-19 pandemic.

Thus, health literacy, whether related to COVID-19 or not, plays an important role in the response to COVID-19. In Japan (as in other countries), fake news and information without scientific basis (such as “The new coronavirus is very heat-sensitive and can be cured by drinking hot water” and “There is a shortage of toilet paper due to the shortage of masks”) have been circulated on the internet and social media, highlighting the importance of health literacy [18]. In addition to broadcasting the number of new COVID-19 infections and deaths, the news media in Japan have broadcast daily pandemic-related information on issues, such as whether health or economy should be prioritized, an increase in bankruptcies and suicides owing to job loss, whether the Tokyo Olympics should be held, which factors have delayed vaccine development and inoculation, and whether government and administrative responses have been inadequate. During a pandemic, there is little time to improve health literacy because governments and citizens must act immediately; therefore, the challenge is to prepare individuals and society for a situation that requires immediate response and containment [1,19].

It is likely that, in Japan, the response to COVID-19 is related to health literacy, but no studies have investigated this. Individual preventive behaviors are important in responding to COVID-19. According to the World Health Organization [20] and the Japanese Ministry of Health, Labor, and Welfare [21], such behaviors include avoiding contact with the virus by maintaining social distancing, wearing masks, and disinfecting hands. Furthermore, to avoid infecting others, individuals with a high temperature or COVID-19 symptoms should rest and stay at home. Theoretically, individuals with greater health literacy should find it easier to obtain relevant health-related information and make appropriate decisions about these behavioral choices; however, health literacy depends, not only on individual ability, but also, on interactions between individuals and the environment. Therefore, the environment must also be conducive to making appropriate behavioral choices. Individuals may find it more difficult to obtain information and make decisions in response to a new disease because the environmental context is more uncertain.

A recent study [22] in Japan demonstrated that comprehensive health literacy is associated with skills in evaluating the reliability of information and decision-making (skills that are not necessarily limited to information about health). In particular, health literacy and the skills to properly evaluate the reliability of new, uncertain, and rapidly changing information (including political and socioeconomic aspects) and to make decisions are required to cope with pandemics and infodemics. The World Health Organization [23] defines an infodemic as “too much information including false or misleading information in digital and physical environments during a disease outbreak.” It would be useful to understand the extent to which information evaluation and decision-making skills are required and which skills are needed to enable individuals to be prepared and respond to emergency situations such as the COVID-19 pandemic. Therefore, the purpose of this study was to determine the extent to which COVID-19 preventive behaviors in Japanese individuals are related to health literacy and to the skills to evaluate and make decisions based on general information from the media (eg, the internet, television, and newspapers).

## Methods

### Participants

Participants were recruited from individuals registered with a Japanese internet research company (Nippon Research Center Ltd) that, as of the time of this study, had approximately 1.4 million voluntarily registered participants. We aimed to collect data from a minimum of 4000 individuals aged 20 to 69 years. In January 2021, potential respondents (n=22,115) were randomly selected and invited via email to participate in a cross-sectional web-based anonymous questionnaire.

In determining potential participants, we tried to match participants’ genders, age groups, and regions (we divided the country into 8 regions) to the results of the 2015 Japanese census [24]. We accepted emailed responses from potential participants until we reached the target number for gender, age group, and region.

### Measures

#### COVID-19 Preventive Behaviors

The questions on COVID-19 preventive behaviors were developed using World Health Organization [20] and Japanese Ministry of Health, Labor, and Welfare [21] guidelines on preventing infection.

To ensure that the questions were as comprehensible as possible, we selected text from easy-to-understand recommendations written for citizens on Japanese government and administrative webpages.

These items were “Use a mask, tissue, handkerchief, or sleeve to cover your mouth and nose when coughing or sneezing,” “Wear a mask when the distance between people is likely to be less than 2 m (meters),” “Wash your hands with soap or alcohol-based disinfectant before meals or upon returning home from outside, etc,” “Try maintaining a minimum distance of 2 m (meters) from people,” “Rest if you are not feeling well,” “Ventilate the room,” “Avoid touching your eyes, mouth, or nose after contact with doorknobs, railings, desks, light switches, etc,” and “Take your temperature.” A 5-point scale (5, always; 4, often; 3, sometimes; 2, rarely; 1, never) was used for response options (see Multimedia Appendix 1). The total score was calculated; higher scores indicated greater frequency of engaging in COVID-19 preventive behaviors.

#### European Health Literacy Survey Questionnaire

The Japanese-language version of the European Health Literacy Survey Questionnaire (HLS-EU-Q47), which is a comprehensive, concept-based measure of most aspects of health literacy for the general population that allows for national and international comparisons [12,25-27], has been used and validated in Japan and in other Asian countries [26,27]. The HLS-EU-Q47 comprises 47 items assessing 12 subdomains of health literacy formed by 4 information processing competences of individuals (accessing, understanding, appraising, and applying) and 3 health contexts (health care, disease prevention, and health promotion). The survey response categories were all phrased similarly to “On a scale from very easy to very difficult, how easy would you say it is to understand why you need health screenings?” and were ranked on a 4-point scale (1, very difficult; 2, fairly difficult; 3, fairly easy; 4, very easy) and included the response option “don’t know/not applicable”; this response was coded as a missing value.

The health literacy score was standardized for each participant on a metric between 0 and 50 using the formula [12]: (*MEAN* – 1) × (50 / 3), where *MEAN* is the mean of all item responses.

#### Information Evaluation

Based on 5 criteria (accuracy, authority, objectivity, currency, and coverage) for judging the quality of information sources [28-32], 5 items were used to determine whether participants were able to evaluate the information [22]. We asked respondents to rate how often they checked the following aspects of the information they accessed on the internet, television, newspapers, magazines, or other media: (1) the source of the information, (2) the qualifications of the people and organizations providing the information, (3) whether the information advertised products or services, (4) when the information was created, and (5) how the information differed from other information. A 5-point scale (5, always; 4, often; 3, sometimes; 2, rarely; 1, never) was used for response options (see Multimedia Appendix 2). Total and item scores were calculated. The internal consistency reliability was excellent (Cronbach α=.92) and construct validity was demonstrated by the results of a confirmatory factor analysis, which produced a single factor. Higher scores indicated greater information evaluation frequency and skill.

#### Decision-making Process

We assessed whether the essential aspects of the process of determining all the available options, knowing the pros and cons of each option, comparing them based on values and preferences, and making a choice were implemented, which is necessary for informed decision-making. For this purpose, we developed 4 items for each aspect based on the Shared Decision-Making Process scale [33]. Items on this scale are limited to 2 options; therefore, we created items that were not limited to health decisions and had a wider range of options. We asked respondents to rate how often they implemented the following aspects when they made important decisions: (1) make sure they have all the options, (2) know the pros of each option, (3) know the cons of each option, and (4) compare the pros and cons of each option and clarify what is important to them. As with the information evaluation items, we used a 5-point scale (5, always; 4, often; 3, sometimes; 2, rarely; 1, never; see Multimedia Appendix 2). Total and item scores were calculated; higher scores indicated greater decision-making frequency and skill. The reliability was excellent (Cronbach α=.93), and construct validity was demonstrated with confirmatory factor analysis, which produced a single factor [22].

#### Demographic Characteristics

The following demographic characteristics were analyzed: gender, age, level of education, occupation, and prefecture status (under a state of emergency or not under a state of emergency). At the time of the survey, the Japanese government had declared a state of emergency because of the COVID-19 pandemic in 11 of the 47 prefectures, including the Tokyo metropolitan area [34]. The main points of this state of emergency plan were shortening the opening hours of restaurants and bars, reducing the number of employees in offices by 70%, avoiding nighttime outings, and limiting events.

### Statistical Analysis

We examined the distribution of responses to each COVID-19 preventive behavior items. Reliability and validity were verified, and Cronbach α values were calculated to examine internal consistency. For construct validity, confirmatory factor analysis was conducted to examine construct validity; the comparative fit index, the root mean square error of approximation, and the standardized root mean square residual were used as model fit indices. A comparative fit index value ≥.95 represents a good fit, and a value ≥.90 is generally considered to indicate acceptable model fit [35,36]. Root mean square error of approximation and standardized root mean square residual values <.05 represent good fits, and values <.08 are acceptable [35,36].

To determine which participants scored higher on COVID-19 preventive behaviors, we conducted multiple linear regression analysis (general linear model) with this variable as the dependent variable and demographic characteristics (gender, age group, education, occupation, and prefecture status) as independent variables.

To determine the extent to which the variables *health literacy*, *information evaluation*, and *decision-making process* can independently explain COVID-19 preventive behaviors, we conducted hierarchical multiple linear regression analysis with the COVID-19 preventive behavior score as the dependent variable and scores on health literacy, information evaluation, and decision-making process as independent variables; demographic characteristics (gender, age group, education, occupation, and prefecture status) were used as control variables.

Data were analyzed using SPSS and Amos software (version 27.0; IBM Corp).

### Ethics Approval and Consent to Participate

The study received prior approval from the Research Ethics Committee of St. Luke’s International University, Japan (20-A076) and was conducted in accordance with the guidelines of the Declaration of Helsinki. Participants voluntarily signed a web-based informed consent form that was approved by the institutional review board.

## Results

### Participants

There were 3914 valid responses (Table 1). These included responses with less than 20% of missing values on all health literacy items, which enabled health literacy scores to be calculated as per the original HLS-EU-Q47 survey [12]. Data for these individuals were included in the analysis.

**Table 1:** Characteristics of study participants.

Characteristic			Participants (n=3914) or value
**Gender, n (%)**			
	Men	1953 (49.9)	
	Women	1961 (50.1)	
**Age (years), n (%)**			
	20-29	567 (14.5)	
	30-39	721 (18.4)	
	40-49	891 (22.8)	
	50-59	785 (20.1)	
	60-69	950 (24.3)	
Age, mean (SD)			46.9 (13.6)
**Highest level of education, n (%)**			
	Junior high school	86 (2.2)	
	High school	981 (25.1)	
	2-year college	858 (21.9)	
	College or university	1806 (46.1)	
	Graduate	183 (4.7)	
**Occupation, n (%)**			
	Self-employed	191 (4.9)	
	Managerial and administrative	166 (4.2)	
	Professional and technical	463 (11.8)	
	Other (routine and manual)	1367 (34.9)	
	Part-time	474 (12.1)	
	Homemaker	652 (16.7)	
	Student	131 (3.3)	
	Unemployed	470 (12.0)	
**Prefecture status, n (%)**			
	Under a state of emergency	2387 (61.0)	
	Not under a state of emergency	1527 (39.0)	
Health literacy score, mean (SD)			27.4 (9.4)

### Distribution of Responses

The item that received the highest percentage of *always* responses was cough etiquette (Table 2), followed by wearing a mask when close to someone, and handwashing. The most infrequently performed behavior was temperature taking (*always* response: 853/3914, 21.8%; often response: 717/3914, 18.3%).

The 3 most frequently performed behaviors had mean scores representing *often* (ie, >4): cough etiquette (mean 4.4, SD 1.0), wearing a mask when close to someone (mean 4.3, SD 1.0), and handwashing (mean 4.1, SD 1.2). The mean total preventive behavior score was 30.1 (SD 6.4).

**Table 2:** Responses for COVID-19 preventive behavior items.

Items	Responses, n (%)						Score, mean (SD)
	Always	Often	Sometimes	Rarely	Never		
1. Use a mask, tissue, handkerchief, or sleeve to cover your mouth and nose when coughing or sneezing	2408 (61.5)	890 (22.7)	368 (9.4)	158 (4.0)	90 (2.3)	4.4 (1.0)	
2. Wear a mask when the distance between people is likely to be less than 2 m (meters)	2234 (57.1)	992 (25.3)	418 (10.7)	162 (4.1)	108 (2.8)	4.3 (1.0)	
3. Wash your hands with soap or alcohol-based disinfectant before meals or upon returning home from outside, etc	2033 (51.9)	930 (23.8)	489 (12.5)	266 (6.8)	196 (5.0)	4.1 (1.2)	
4. Try maintaining a minimum distance of 2 m (meters) from people	845 (21.6)	1479 (37.8)	986 (25.2)	440 (11.2)	164 (4.2)	3.6 (1.1)	
5. Rest if you are not feeling well	1217 (31.1)	1059 (27.1)	805 (20.6)	497 (12.7)	336 (8.6)	3.6 (1.3)	
6. Ventilate the room	1037 (26.5)	1133 (28.9)	986 (25.2)	506 (12.9)	252 (6.4)	3.6 (1.2)	
7. Avoid touching your eyes, mouth, or nose after contact with doorknobs, railings, desks, light switches, etc	1035 (26.4)	1111 (28.4)	857 (21.9)	495 (12.6)	416 (10.6)	3.5 (1.3)	
8. Take your temperature	853 (21.8)	717 (18.3)	932 (23.8)	798 (20.4)	614 (15.7)	3.1 (1.4)	

### Reliability and Validity of the Total COVID-19 Preventive Behavior Score

We confirmed the reliability and validity of the total COVID-19 preventive behavior score (Cronbach α=.83). The comparative fit index was 0.963, the root mean square error of approximation was 0.073 (95% CI 0.067- 0.079), and the standardized root mean square residual was 0.035, which indicated acceptable fit. Error covariances were observed between 2 sets of items with similar wording in Japanese (set 1: items 1 and 2, set 2: items 5 and 8), but confirmatory factor analysis factor loadings were >0.45 for all items, and a unidimensional structure was confirmed.

### Multiple Linear Regression

For COVID-19 preventive behaviors, scores were higher for women than for men (*P*<.001), and participants with higher levels of education had higher scores (*P*<.001) (Table 3). Participants in occupational, managerial, and administrative jobs had the highest preventive behavior scores, and participants who were unemployed had the lowest scores (*P*<.001). Participants in prefectures under a state of emergency had higher preventive behavior scores (*P*<.001).

**Table 3:** Multiple linear regression results for COVID-19 preventive behaviors as the dependent variable.

Variables		Estimated marginal mean (95% CI)	*F* test (*df*)	*P* value
**Gender**			141.9 (1,3913)	<.001
	Men	28.4 (27.9, 28.8)		
	Women	31.2 (30.7, 31.7)		
**Age (years)**			.7 (4,3913)	.63
	20-29	29.7 (29.1, 30.3)		
	30-39	29.9 (29.4, 30.5)		
	40-49	29.6 (29.1, 30.2)		
	50-59	29.6 (29.1, 30.2)		
	60-69	30.0 (29.5, 30.5)		
**Highest level of education**			4.6 (4,3913)	.001
	Junior high school	28.0 (26.6, 29.3)		
	High school	29.7 (29.3, 30.1)		
	2-year college	30.4 (30.0, 30.9)		
	College or university	30.2 (29.9, 30.6)		
	Graduate	30.6 (29.7, 31.6)		
**Occupation**			6.0 (7,3913)	<.001
	Self-employed	29.3 (28.4, 30.3)		
	Managerial and administrative	31.5 (30.5, 32.5)		
	Professional and technical	29.1 (28.5, 29.7)		
	Other (routine and manual)	29.6 (29.2, 30.1)		
	Part-time	29.8 (29.2, 30.4)		
	Homemaker	30.4 (29.8, 31.0)		
	Student	30.0 (28.8, 31.2)		
	Unemployed	28.6 (27.9, 29.2)		
**Prefecture**			33.1 (1,3913)	<.001
	Under a state of emergency	30.4 (30.0, 30.8)		
	Not under a state of emergency	29.2 (28.8, 29.6)		

### Hierarchical Multiple Linear Regression

Pearson correlations (Table 4) between COVID-19 preventive behaviors and health literacy, information evaluation, and decision-making process were *r*=0.23 (*P*<.001), *r*=0.24 (*P*<.001), and *r*=0.30 (*P*<.001), respectively.

An examination of the change in health literacy from model 1, in which only health literacy was entered as an independent variable, to model 3, which included all 3 variables, showed that the standardized regression coefficient approximately halved (model 1: β=.20; model 3: β=.11), whereas the changes for information evaluation (model 2: β=.15; model 3: β=.13) and decision-making process (model 1: β=.20; model 3: β=.18) were less pronounced; both standardized regression coefficient remained similar even after controlling for health literacy.

**Table 4:** Hierarchical multiple linear regression analysis of COVID-19 preventive behaviors, controlling for demographic variables (gender, age, education, occupation, and prefecture status).

Independent variable	Correlation			Model 1^a^				Model 2^b^				Model 3^c^		
	*r*	*P* value	β		*t* value	*P* value	β		*t* value	*P* value	β		*t* value	*P* value
Health literacy	0.23	<.001	.20		13.0	<.001	—^d^		—	—	.11		6.8	<.001
Information evaluation	0.24	<.001	—		—	—	.15		8.3	<.001	.13		7.4	<.001
Decision-making process	0.30	<.001	—		—	—	.20		11.3	<.001	.18		9.9	<.001

^a^*R*^2^=0.12, and adjusted *R*^2^=0.12; *F*(18,3913)=30.6, *P*<.001.

^b^*R*^2^=0.18, and adjusted *R*^2^=0.18; *F*(19,3913)=45.3, *P*<.001.

^c^*R*^2^=0.19, and adjusted *R*^2^=0.19; *F*(20,3913)=45.9, *P*<.001.

^d^Data not included.

## Discussion

This study demonstrated that health literacy was associated with COVID-19 preventive behaviors. However, information evaluation and decision-making process skills, which are not limited to health information, showed a similar strength of association with preventive behaviors. Because the assessment of health literacy in this survey focused mainly on daily health information, it may reflect the ability to cope with common diseases, including familiar infectious diseases and may be inadequate to assess responses to pandemics caused by new viruses (or infodemics). In situations in which the evidence is insufficient or not immediately communicated in an easy-to-understand manner, skills in information evaluation and decision-making process (not necessarily limited to health information) are important.

The decision-making process showed the strongest association with preventive behaviors in all analyses, even after controlling for health literacy and information evaluation. The results show that an engagement in COVID-19 preventive behaviors is associated with rational decision-making skills. To make rational decisions (ie, decisions that are purposeful and have clear reasons), an individual must engage in the process of generating options, comparing the pros and cons of those options, and selecting the option that best fits their values. The importance of this process has been demonstrated in business and health, both of which require individuals to regularly make important decisions [37]. This process is essential to the practice of shared decision-making in health, which is a collaboration between health care professionals and consumers [38]. Similarly, in evidence-based health care, consumer values and decision-making preferences are as important as evidence [39]. Furthermore, the Ottawa Decision Support Framework [40] states that to improve the quality of decision-making, individuals must make choices according to the perceived importance of the pros and cons of each option.

To implement COVID-19 preventive behaviors, we first need to know what the options are and which behaviors lower or raise the risk. Then, we need to be able to evaluate the balance and trade-offs between infection-prevention behaviors and work, connections with family and friends, stress, and mental health. To make decisions appropriate to their personal values, people need to be familiar with making decisions that clarify their values on a regular basis; it may be difficult to clarify personal values on the spur of the moment. Some international comparative studies [41-43] have shown that Japanese people have low self-esteem in decision-making and tend to make intuitive decisions rather than rational decisions. Therefore, this is an opportunity to draw attention to the importance of rational decision-making skills.

The association between preventive behaviors and information evaluation and decision-making skills that we found may indicate that the lack of these skills leads to a higher risk of infection. These differences could result in individual disparities in new health issues, which could be greater for infectious diseases as people infect others around them. In making important decisions, it is necessary to ensure that the information on which the decision is based is sufficiently reliable to determine the pros and cons of each option and to identify which pros and cons are important. However, in a survey [22] in Japan, only approximately 30% to 50% of respondents answered that they always or often made decisions in such a manner. Furthermore, more than 40% of respondents reported that they did not have the opportunity to learn these skills. Therefore, it is necessary to create an environment in which everyone can learn these skills. Respondents who had had the opportunity to learn these skills usually acquired them from the internet (approximately 40%), followed by television (approximately 30%), and newspapers and magazines (approximately 15%). It may therefore be useful to consider providing information through these media [22].

Our findings also indicate that individuals who lack skills in information evaluation and decision-making do not receive sufficient reliable information to enable decision-making. Transparent, honest communication is important to control the pandemic [4]. It would be useful to develop a website or social media source where people could obtain the latest reliable and easy-to-understand information and make decisions. In Japan, there is no organization equivalent to the US Centers for Disease Control and Prevention, and no clear source of information in the event of an infectious disease or pandemic; therefore, a solution to this problem is needed.

In addition to providing information and services that are easy to understand, there is a need to support decision-making, especially for individuals with poor decision-making skills. One early initiative was the creation of a decision aid by the Gerontological Society of America to enable people to determine whether to interact with people and participate in activities outside the home [44]. To prepare for pandemics, it is necessary to create a system that can rapidly develop and disseminate such a tool.

This study had several limitations. It is possible that there was some sample selection bias. Participants may have been skewed toward a high level of internet literacy because of the use of a web-based survey. Recruitment of respondents was based on self-selection from a group of individuals who had previously expressed a desire to participate in research projects. The responses were limited to approximately the first 4000 people; therefore the sample may have included only individuals who were most active on the internet (eg, frequently checking email). Although users familiar with the internet and social media may find it easier to obtain health information, they may become confused by the large amount of often contradictory health information available. There is evidence that internet literacy is not the only factor that determines whether people access health information using electronic sources [45]. The results of our study suggest that even people with sufficient internet literacy also need health literacy, information evaluation skills, and decision-making skills to take appropriate health action in response to an infodemic.

Items were created from 5 information evaluation criteria; however, these were just representative items; many alternative, more detailed items could have been chosen (eg, the affiliation of the author of the information). However, rather than covering a wide range of content, the goal was to identify the core aspects of information evaluation associated with COVID-19 preventive behaviors. These issues also apply to the 4 decision-making items. For example, the description *decision-making* includes clarification of the problem before checking the options, as well as action and evaluation after the decision is made. However, because we used the shared decision-making process as a reference, we focused on the process required to make a decision, assuming that the problem was already apparent. The aim was, not to create a scale to cover all skills needed in the decision-making process, but, to determine if specific key points were related to COVID-19 preventive behaviors.

This study was also limited because it used cross-sectional data, which does not allow a firm conclusion to be drawn.

For new nonroutine health challenges, for example, understanding and using preventive behaviors during a pandemic such as that of COVID-19, the ability to evaluate all information and make appropriate decisions is required. However, because some people experience difficulty with this, there is a substantial need to provide reliable and easy-to-understand information and to support people in choosing appropriate actions by creating an environment that allows individuals to learn information evaluation and decision-making skills at any age.

## Appendix

Multimedia Appendix 1 Questionnaire on COVID-19 preventive behaviors (Japanese version).

Multimedia Appendix 2 Questionnaire on information evaluation and decision-making process skills (Japanese version).

## Abbreviations

HLS-EU-Q47: European Health Literacy Survey Questionnaire
