# Supplementary material for: COVID-19 Preventive Behaviors and Health Literacy, Information Evaluation, and Decision-making Skills in Japanese Adults: Cross-sectional Survey Study
Source: JMIR Form Res. 2022 Jan 24;6(1):e34966. doi: 10.2196/34966 (PMC8822428; doi:10.2196/34966)
Supplement: Multimedia Appendix 1 [file formative_v6i1e34966_app1.docx]

Multimedia Appendix 1. Questionnaire on Covid-19 preventive behaviors (Japanese version)

Covid-19予防行動の質問紙 (日本語版)

あなたは、次のようなことをしていますか。それぞれ「いつもしている」から「まったくしていない」までで、最もあてはまるものを選択してください。（それぞれひとつずつ）

|  | いつも  している | よく  している | ときどき  している | たまに  している | まったく  していない |
| --- | --- | --- | --- | --- | --- |
| 咳・くしゃみをするときは、マスクやティッシュ・ハンカチ、袖を使って、口や鼻をおさえる | １ | ２ | ３ | ４ | ５ |
| 人と人との間が２ｍ（メートル）未満になりそうな時はマスクをする | １ | ２ | ３ | ４ | ５ |
| 外出先からの帰宅後、食事前などにせっけん・アルコール消毒液で手を洗う | １ | ２ | ３ | ４ | ５ |
| 人と人との間は、できる限り２m（メートル）空ける | １ | ２ | ３ | ４ | ５ |
| 体調が悪いときは休む | １ | ２ | ３ | ４ | ５ |
| 部屋の換気をする | １ | ２ | ３ | ４ | ５ |
| ドアノブや手すり、机、スイッチなどに触れた後、目や口や鼻を触らない | １ | ２ | ３ | ４ | ５ |
| 体温を測る | １ | ２ | ３ | ４ | ５ |

作成代表者　中山和弘（聖路加国際大学大学院看護学研究科）　nakayama@slcn.ac.jp
