# Supplementary material for: COVID-19 Preventive Behaviors and Health Literacy, Information Evaluation, and Decision-making Skills in Japanese Adults: Cross-sectional Survey Study
Source: JMIR Form Res. 2022 Jan 24;6(1):e34966. doi: 10.2196/34966 (PMC8822428; doi:10.2196/34966)
Supplement: Multimedia Appendix 2 [file formative_v6i1e34966_app2.docx]

Multimedia Appendix 2. Questionnaire on information-evaluation and decision-making process skills (Japanese version)

情報の評価と意思決定プロセスの質問紙（日本語版）

あなたはインターネット、テレビ、新聞、雑誌などのメディアの情報を見るとき、次にあげることをそれぞれどのくらい確認していますか。「いつもしている」から「まったくしていない」までで、最もあてはまるものを選択してください。（それぞれひとつずつ）

|  | いつも  している | よく  している | ときどき  している | たまに  している | まったく  していない |
| --- | --- | --- | --- | --- | --- |
| 情報はいつ作られたものか | １ | ２ | ３ | ４ | ５ |
| 情報は、商品やサービスの宣伝のためではないか | １ | ２ | ３ | ４ | ５ |
| 情報を出している人や団体は、どのような資格を持つ人たちか | １ | ２ | ３ | ４ | ５ |
| 情報の元ネタ（情報源）は何か | １ | ２ | ３ | ４ | ５ |
| 別の情報と比べてどのような違いがあるか | １ | ２ | ３ | ４ | ５ |

あなたは大事なことを決める時に、次のようなことをしていますか。それぞれ「いつもしている」から「まったくしていない」までで、最もあてはまるものを選択してください。 （ぞれぞれひとつずつ）

|  | いつも  している | よく  している | ときどき  している | たまに  している | まったく  していない |
| --- | --- | --- | --- | --- | --- |
| 選べる選択肢がすべてそろっているか確認する | １ | ２ | ３ | ４ | ５ |
| 各選択肢の長所を知る | １ | ２ | ３ | ４ | ５ |
| 各選択肢の短所を知る | １ | ２ | ３ | ４ | ５ |
| 各選択肢の長所と短所を比較して、自分にとって何が重要かはっきりさせる | １ | ２ | ３ | ４ | ５ |

作成代表者　中山和弘（聖路加国際大学大学院看護学研究科）　nakayama@slcn.ac.jp
